# Supplementary material for: Inflammation-induced Id2 promotes plasticity in regulatory T cells
Source: Nat Commun. 2018 Nov 9;9:4736. doi: 10.1038/s41467-018-07254-2 (PMC6226514; doi:10.1038/s41467-018-07254-2)
Supplement: Supplementary file 1 — Supplementary Information [file 41467_2018_7254_MOESM1_ESM.pdf]

Supplementary information

Inflammation-induced Id2 promotes plasticity in regulatory T cells

Hwang et al.

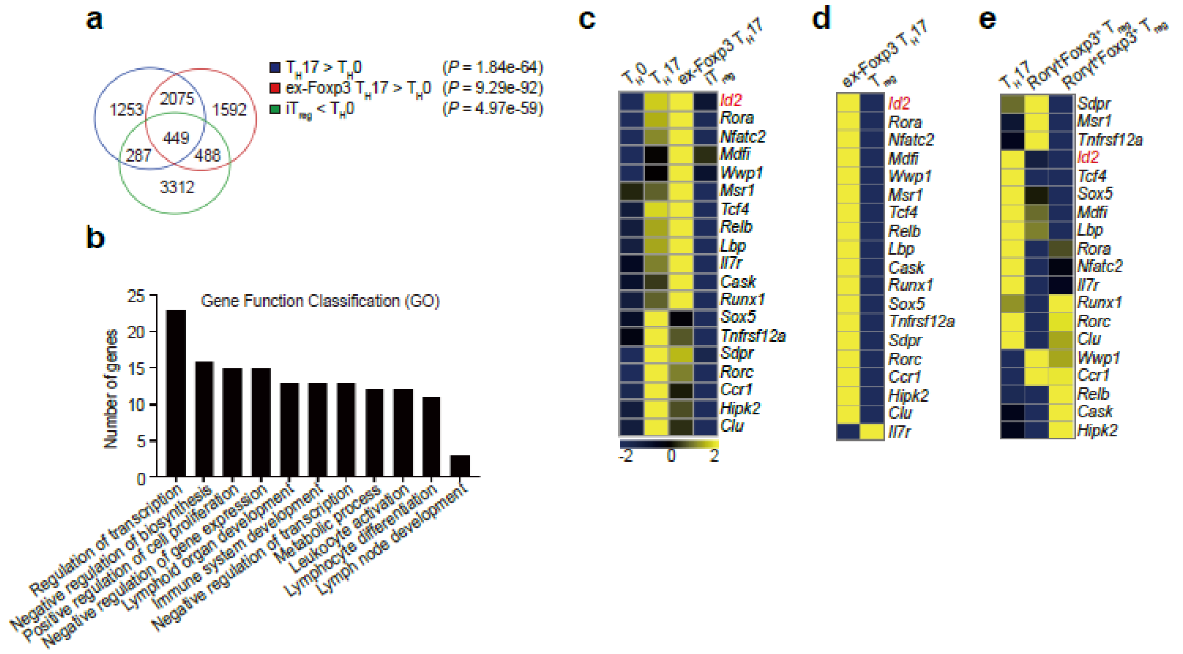

Supplementary Figure 1

Id2 is highly expressed in ex-Foxp3 T_H17 cells.

a Microarray analysis (GEO accession code GSE48428) representing overlap of differentially expressed genes more in ex-Foxp3 T_H17 cells and T_H17 cells and less in iT_{reg} cells than T_H0 cells (2-fold). **b** Gene Ontology (GO) analysis on 11 significant GO terms associated with 449 genes in which were normally suppressed by Foxp3 expression in iT_{reg} cells while induced as absence of Foxp3 expression in ex-Foxp3 T_H17 cells and T_H17 cells. **c** Heat map of selected 19 target genes related to immune system and /or are involved in regulation of transcription from (**b**). **d,e** Validation of selected 19 target genes expression from two other independent studies (GEO accession code GSE80804;**d**, GSE60059;**e**).

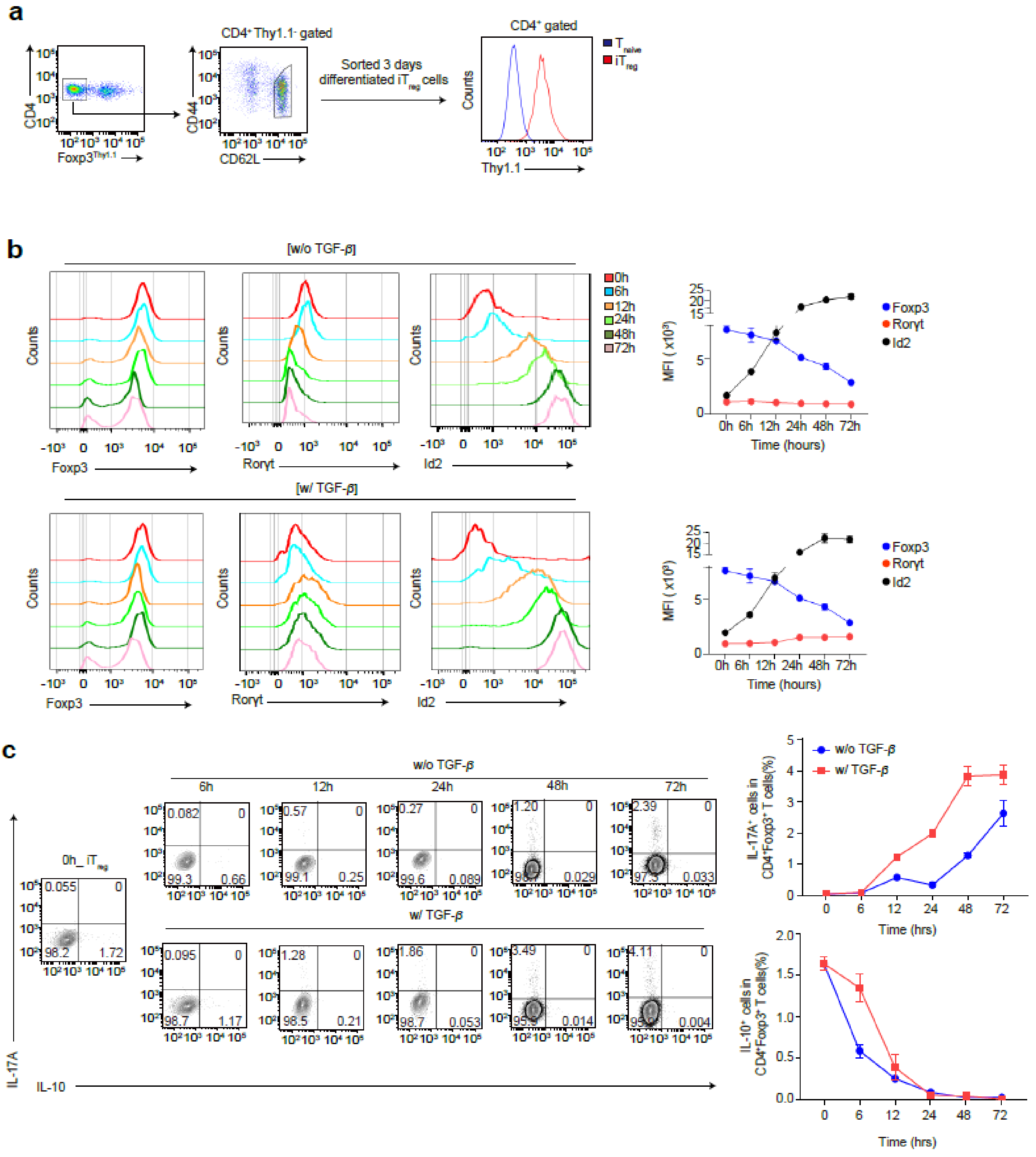

Supplementary Figure 2

Kinetics of iT$_{reg}$ - ex-Foxp3 T$_H$17 conversion.

a Strategy for sorting naïve CD4$^+$ T cells and differentiated iT$_{reg}$ cells from Foxp3$^{Thy1.1}$ reporter mice. **b,c** Sorted CD4$^+$CD62L^{hi}CD44^{lo}Thy1.1$^-$ naïve T cells were activated *in vitro* under iT$_{reg}$ cell differentiation conditions for 3 days. After 3 days, iT$_{reg}$ cells were re-stimulated *in vitro* in ex-Foxp3 T$_H$17 polarizing conditions with(w/) or without(w/o) exogenous TGF-β for additional 3 days. Representative FACS plot showing expression of Foxp3, Rorγt, Id2 (**b**), IL-10 and IL-17A (**c**) positive populations in CD4$^+$Foxp3$^+$ T cells after harvesting cells at indicated time points (Data is summary of three experiments).

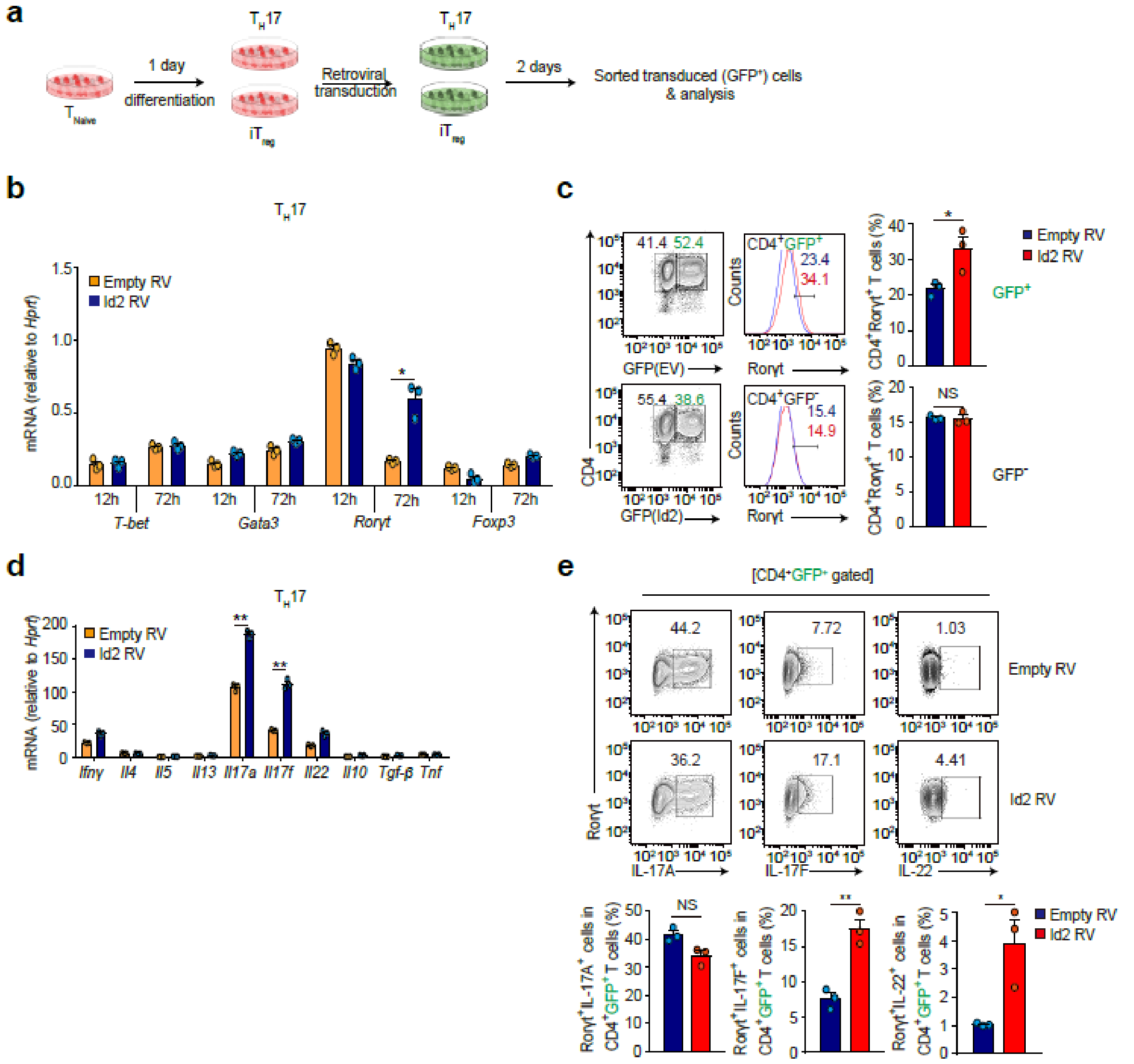

Supplementary Figure 3

Effect of Id2 overexpression during T$_H$17 and iT$_{reg}$ differentiation conditions.

a A Schematic of retroviral transduction of naïve CD4$^+$ T cells. Naïve CD4$^+$ T cells that were activated *in vitro* were transduced with Empty-GFP RV or Id2-GFP RV on day 1 under each T helper (T$_H$17 and iT$_{reg}$) cell-differentiation conditions. The following day 2 after culture, GFP$^+$ cells were sorted and analyzed. **b** RT-qPCR analysis of *T-bet, Gata3, Rorγt* and *Foxp3* mRNA expression between Empty RV and Id2 RV transduced T cells on day 3 post-infection during T$_H$17 cell-differentiation conditions (n=3-5, per group). **c** Flow cytometry analysis of Rorγt expression in Empty RV or Id2 RV transduced (GFP$^+$) and non-transduced (GFP$^-$) T$_H$17 cells. **d** RT-qPCR analysis of the indicated cytokine encoding mRNA expression in Empty RV and Id2 RV transduced T cells after day 3 post-infection during iT$_{reg}$ cell-differentiation conditions. **e** Flow cytometry analysis of IL-17A, IL-17F and IL-22 from Empty RV or Id2 RV transduced CD4$^+$GFP$^+$ T$_H$17 cells. NS=Non-Significant, * $P < 0.05$, ** $P < 0.005$ (Student's *t*-test). Data are representative two to three independent experiments (error bars, s.d.).

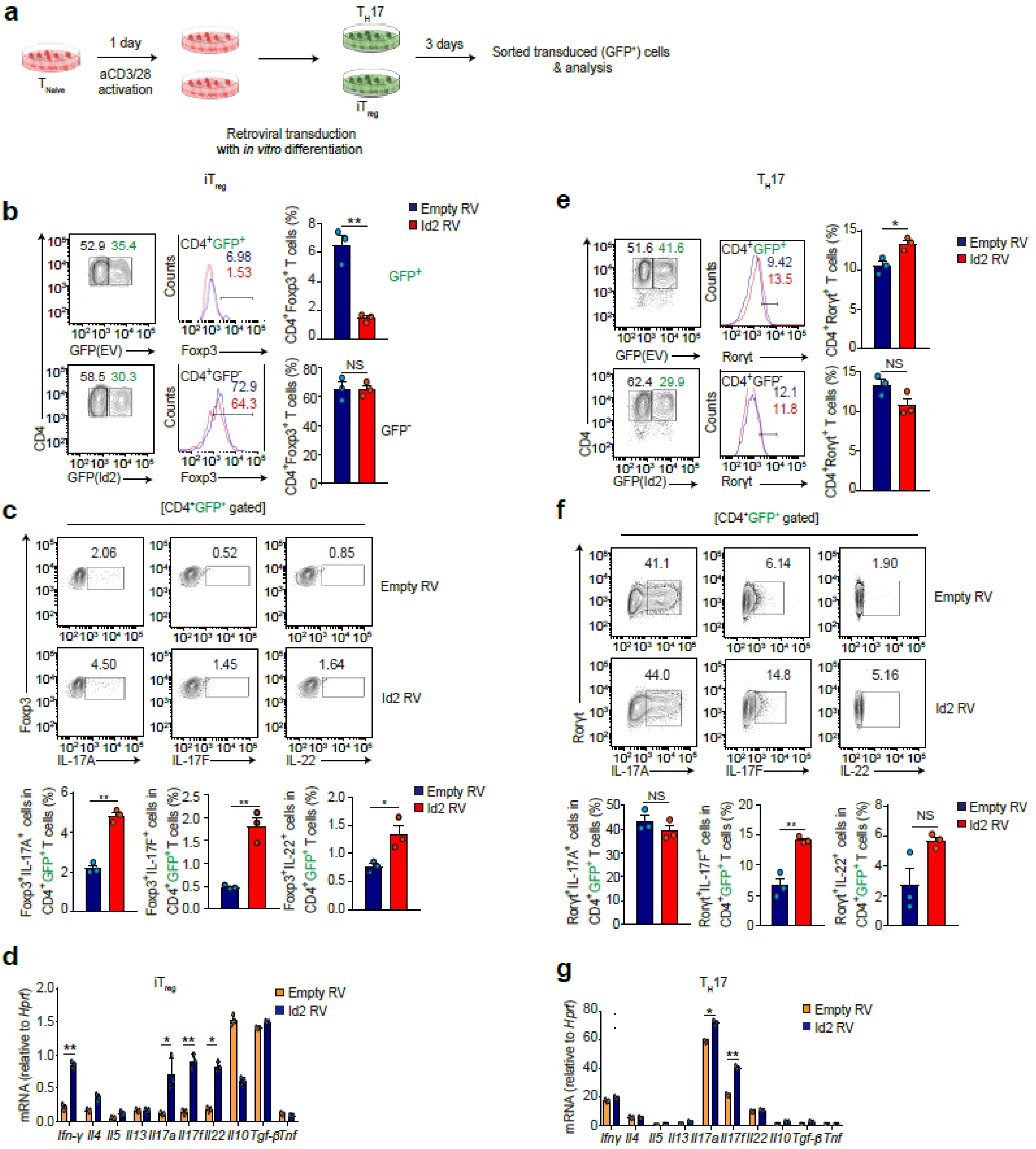
a
1 day
aCD3/28 activation
T_{Naive}
T_H17
iT_{reg}
3 days
Sorted transduced (GFP^+) cells & analysis
Retroviral transduction with *in vitro* differentiation
b
iT_{reg}
52.9 35.4
58.5 30.3
GFP(EV)
GFP(Id2)
CD4
$CD4^+GFP^+$
6.98
1.53
$CD4^+GFP^-$
72.9
64.3
Counts
Foxp3
$CD4^+Foxp3^+$ T cells (%)
GFP^+
GFP^-
Empty RV
Id2 RV
NS
c
[$CD4^+GFP^+$ gated]
2.06
0.52
0.85
4.50
1.45
1.64
Empty RV
Id2 RV
Foxp3
IL-17A
IL-17F
IL-22
$Foxp3^+IL\text{-}17A^+$ cells in $CD4^+GFP^+$ T cells (%)
$Foxp3^+IL\text{-}17F^+$ cells in $CD4^+GFP^+$ T cells (%)
$Foxp3^+IL\text{-}22^+$ cells in $CD4^+GFP^+$ T cells (%)
d
iT_{reg}
mRNA (relative to *Hprt*)
Ifn-γ *Il4* *Il5* *Il13* *Il17a* *Il17f* *Il22* *Il10* *Tgf-β* *Tnf*
e
T_H17
51.6 41.6
62.4 29.9
GFP(EV)
GFP(Id2)
CD4
$CD4^+GFP^+$
9.42
13.5
$CD4^+GFP^-$
12.1
11.8
Counts
Rorγt
$CD4^+Ror\gamma t^+$ T cells (%)
f
[$CD4^+GFP^+$ gated]
41.1
6.14
1.90
44.0
14.8
5.16
Rorγt
IL-17A
IL-17F
IL-22
$Ror\gamma t^+IL\text{-}17A^+$ cells in $CD4^+GFP^+$ T cells (%)
$Ror\gamma t^+IL\text{-}17F^+$ cells in $CD4^+GFP^+$ T cells (%)
$Ror\gamma t^+IL\text{-}22^+$ cells in $CD4^+GFP^+$ T cells (%)
g
T_H17
mRNA (relative to *Hprt*)
Ifnγ *Il4* *Il5* *Il13* *Il17a* *Il17f* *Il22* *Il10* *Tgf-β* *Tnf*

Supplementary Figure 4

Effect of Id2 overexpression concomitant to iT_{reg} or T_H17 differentiation.

a Naïve CD4$^+$ T cells were sorted from wild-type C57BL/6 (B6) mice, and transduced with control vector (Empty RV) or vector encoding Id2 cDNA (Id2 RV) after 1 day activated by plate bound anti-CD3/28. Cells were then differentiated under iT_{reg} or T_H17 differentiation conditions. After 3 days cells were harvested and Id2 expression was measured by RT-qPCR. **b,c** Flow cytometry analysis of Foxp3 **(b)** expression and IL-17A, IL-17F and IL-22 **(c)** between Empty RV or Id2 RV transduced (GFP$^+$) and non-transduced (GFP$^-$) iT_{reg} cells. **d,** RT-qPCR analysis of mRNA expression for the indicated cytokines in Empty RV and Id2 RV transduced T cells sorted after 3 days post-infection followed by iT_{reg} cell-differentiation. **e,f** Flow cytometry analysis of Rorγt **(e)** expression and IL-17A, IL-17F and IL-22 **(f)** in Empty RV or Id2 RV transduced (GFP$^+$) and non-transduced (GFP$^-$) T_H17 cells. **g** RT-qPCR analysis of the expressions of indicated cytokines in Empty RV and Id2 RV transduced T cells sorted after day 3 post-infection under T_H17 cell-differentiation conditions. NS=Non-Significant, * $P < 0.05$, ** $P < 0.005$ (Student's *t*-test). Data are representative of two independent experiments (error bars, s.d.).

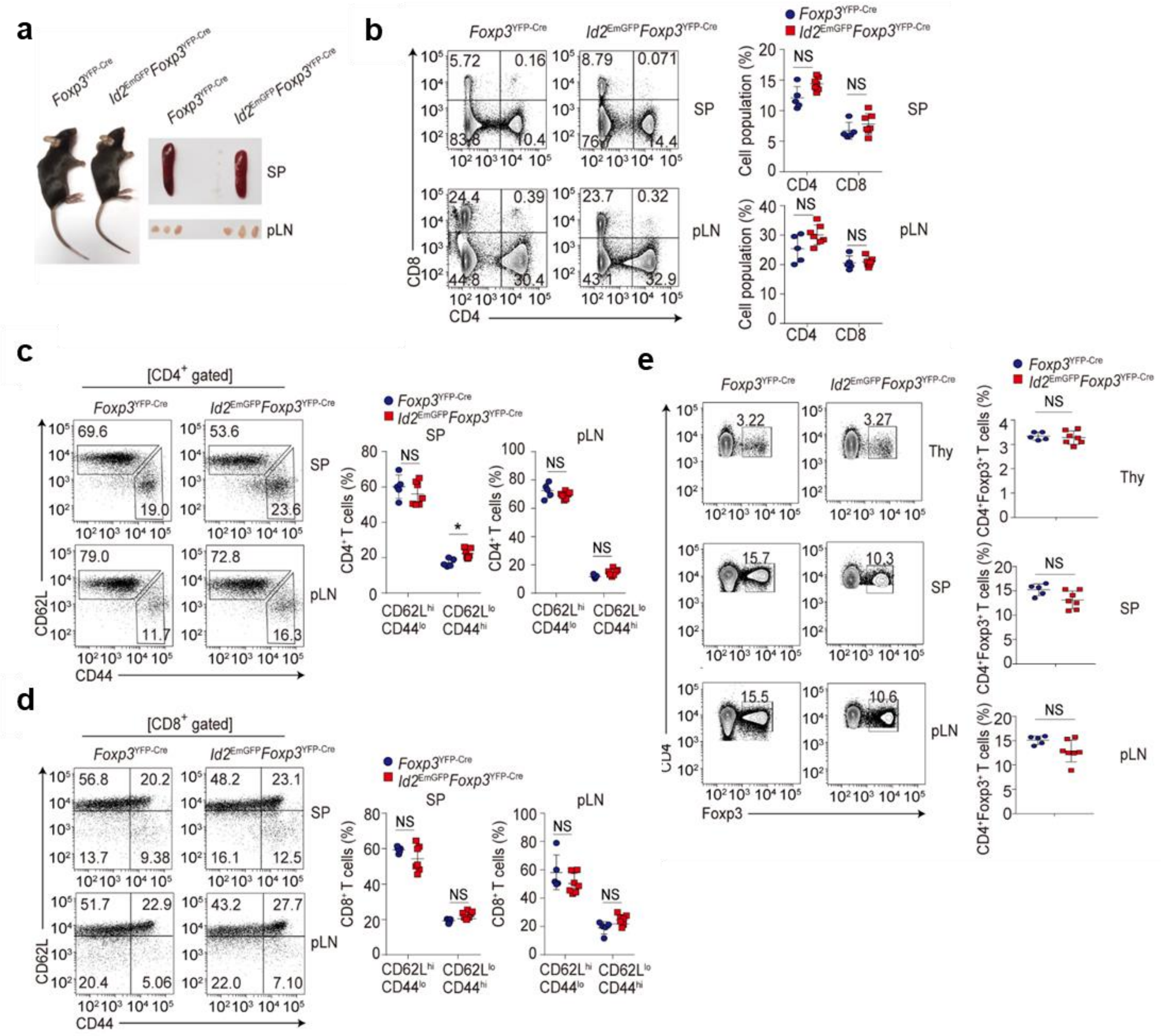

Supplementary Figure 5

Analysis of 6-8 weeks of *Id2*^{EmGFP}*Foxp3*$^{\text{YFP-Cre}}$ mice.

a A representative picture of 6-8 week-old *Foxp3*$^{\text{YFP-Cre}}$ and *Id2*^{EmGFP}*Foxp3*$^{\text{YFP-Cre}}$ mice (*left*) and spleen (SP) and peripheral lymph nodes (pLN) from those mice (*right*). **b** Percentages of CD4^{+} and CD8^{+} T cells from SP and pLN (*Foxp3*$^{\text{YFP-Cre}}$; n=5, *Id2*^{EmGFP}; n=7). **c,d** Percentages of CD62L^{hi}CD44^{lo} (naïve) and CD62L^{lo}CD44^{hi} (effector/memory) cells among CD4^{+} and CD8^{+} T cells in SP and pLN (*Foxp3*$^{\text{YFP-Cre}}$; n=5, *Id2*^{EmGFP}; n=7). **e** Foxp3^{+} cell percentages among CD4^{+} T cells in thymus, SP and pLN from 6-8 week-old mice (*Foxp3*$^{\text{YFP-Cre}}$; n=5, *Id2*^{EmGFP}; n=7). NS=Non-Significant, * $P < 0.05$. All data are representative three independent experiments (error bars, s.d.).

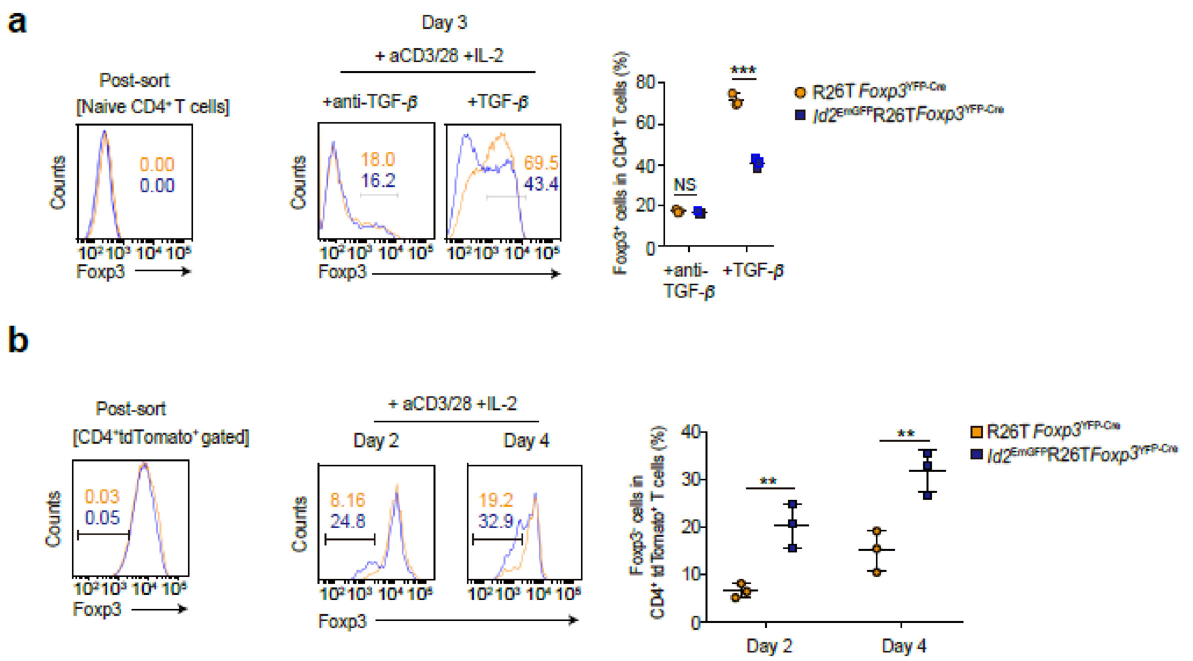

Supplementary Figure 6

T_{reg}-specific ectopic expression of Id2 results in enhanced loss of Foxp3 expression from T_{reg} cells, and not enhanced stability of promiscuous Foxp3 expressing naïve T cells.

a Sorted naïve CD4$^+$ T cells from R26T*Foxp3*$^{\text{YFP-Cre}}$ and *Id2*^{EmGFP}R26T*Foxp3*$^{\text{YFP-Cre}}$ mice were activated with anti-CD3, anti-CD28 and IL-2 in the presence of TGF-β blocking antibody (anti-TGF-β) or TGF-β for 3 days. After 3 days, Foxp3-expression was measured by flow cytometry. **b** Sorted T_{reg} cells (CD4$^+$tdtmomato$^+$YFP$^+$) from R26T*Foxp3*$^{\text{YFP-Cre}}$ and *Id2*^{EmGFP}R26T*Foxp3*$^{\text{YFP-Cre}}$ mice were activated with anti-CD3, anti-CD28 and IL-2 *in vitro*. Expression of Foxp3 was measured by flow cytometry at indicated time points. NS=Non-Significant, ** $P < 0.01$, *** $P < 0.001$, **** $P < 0.0001$. Data are representative of three independent experiments (error bars, s.d.).

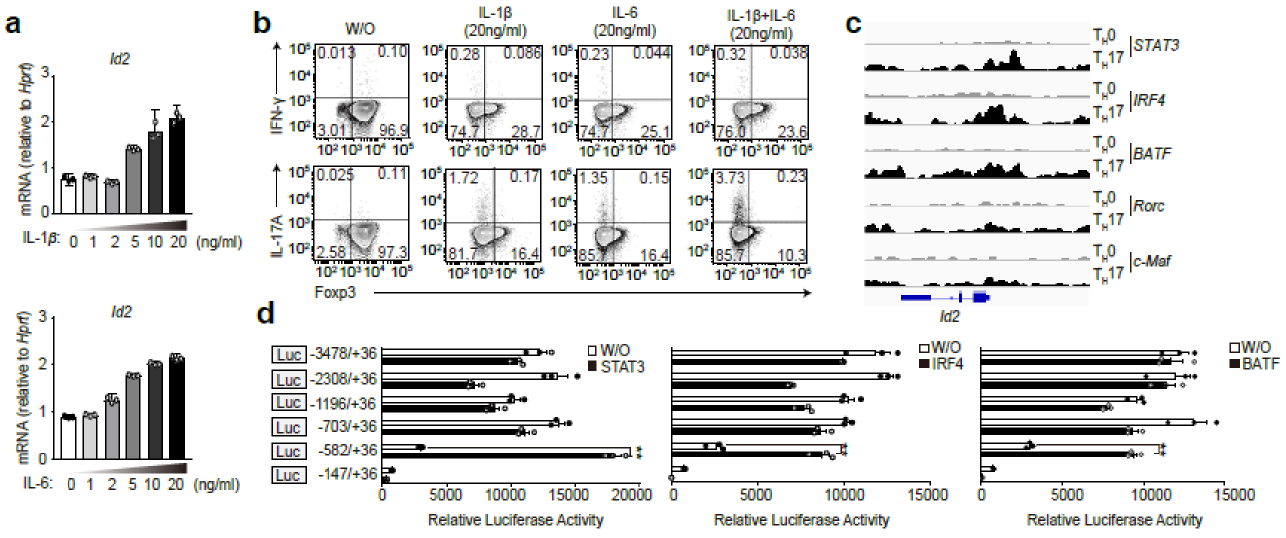

Supplementary Figure 7

Id2 is induced by IL-1β and IL-6 signaling-mediated STAT3, IRF4 and BATF in T_{reg} cells.

a RT-qPCR analysis of *Id2* mRNA expression given at different dose of IL-1β (upper) and IL-6 (lower) in iT_{reg} cells. **b** Intracellular staining of IFN-γ, IL-17A and Foxp3 expression in W/O, IL-1β (20ng/ml), IL-6 (20ng/ml) and IL-1β+ IL-6 (20ng/ml) conditions for 2 days in iT_{reg} cells. **c** ChIP-seq results for STAT3, IRF4 and BATF at the Id2 locus in T_H0 and T_H17 cells (GEO accession code GSM1004793). **d** Analysis of luciferase activity from six serial-deletion of Id2 promoter-luciferase constructs transfected into HEK 293T cells with STAT3, IRF4 and BATF expressing vectors, respectively. (W/O; control). Luciferase activities were measured with the reporter activities normalized to renilla luciferase activity. ** $P < 0.005$ (Student's *t*-test). All data are representative three independent experiments with similar results (error bars, s.d.).

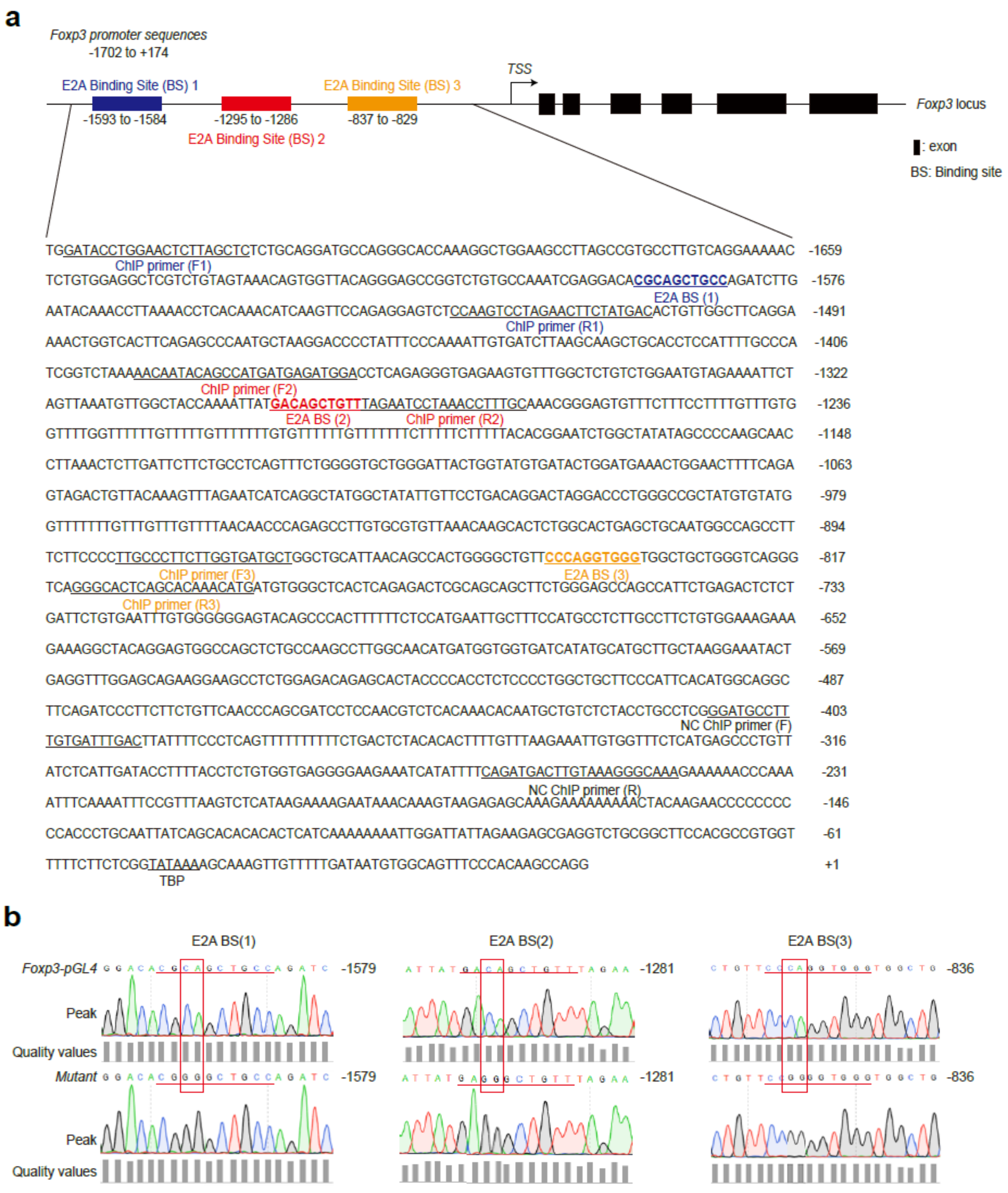

Supplementary Figure 8

Nucleotide sequences of the *Foxp3* promoter and E2A binding sites.

a Sequences of *Foxp3* promoter from -1741 to +1. Highly-putative E2A binding sites (score>10) are marked in blue, red and yellow (BS1, BS2 and BS3, respectively). Each ChIP-primers (F/R) were indicated, respectively. **b** Sequencing results of mutation of E2A binding sites (Mutants; BS1, BS2 and BS3) in the corresponding control (Foxp3-pGL4). The rectangle indicates the nucleotides comprising a major E2A binding sites (E-box site).

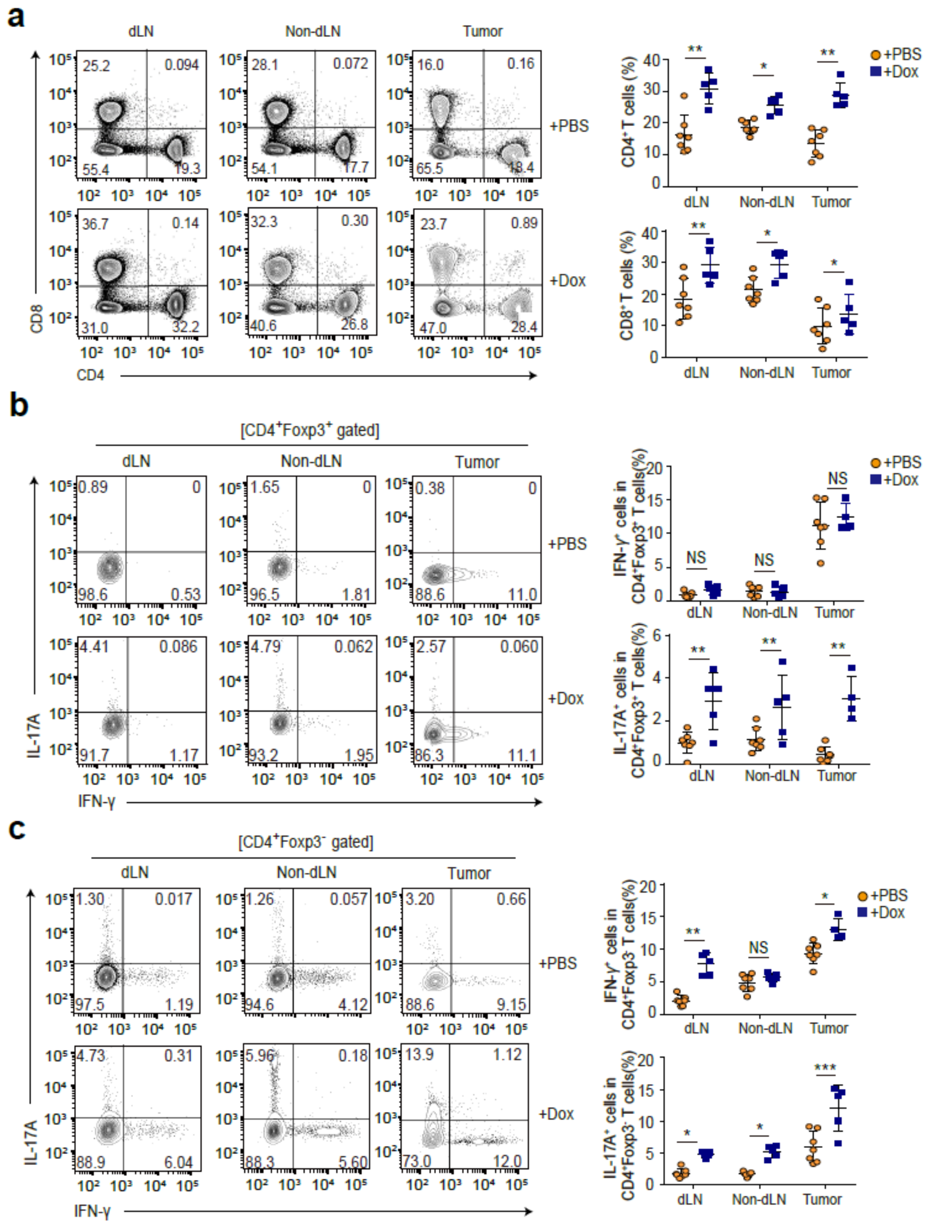

Supplementary Figure 9

Increased T cell infiltration and IL-17A expression in doxycycline-treated TetR-*Id2*^{EmGFP} mice.

a Flow cytometry analysis of CD4$^+$ and CD8$^+$ T cells in tumor-draining lymph nodes (dLN), non-draining lymph nodes(non-dLN) and tumor (PBS; n=7, Dox; n=5). **b,c** Intracellular staining of cytokines in CD4$^+$Foxp3$^+$ and CD4$^+$Foxp3$^-$ T cells isolated from dLN, non-dLN and tumor (PBS; n=7, Dox; n=5), stimulated with phorbol myristate acetate (PMA) and ionomycin for 6 h. NS=Non-Significant, * $P < 0.05$, ** $P < 0.005$, *** $P < 0.001$ (Student's *t*-test). All data are representative of three independent experiments (error bars, s.d.).

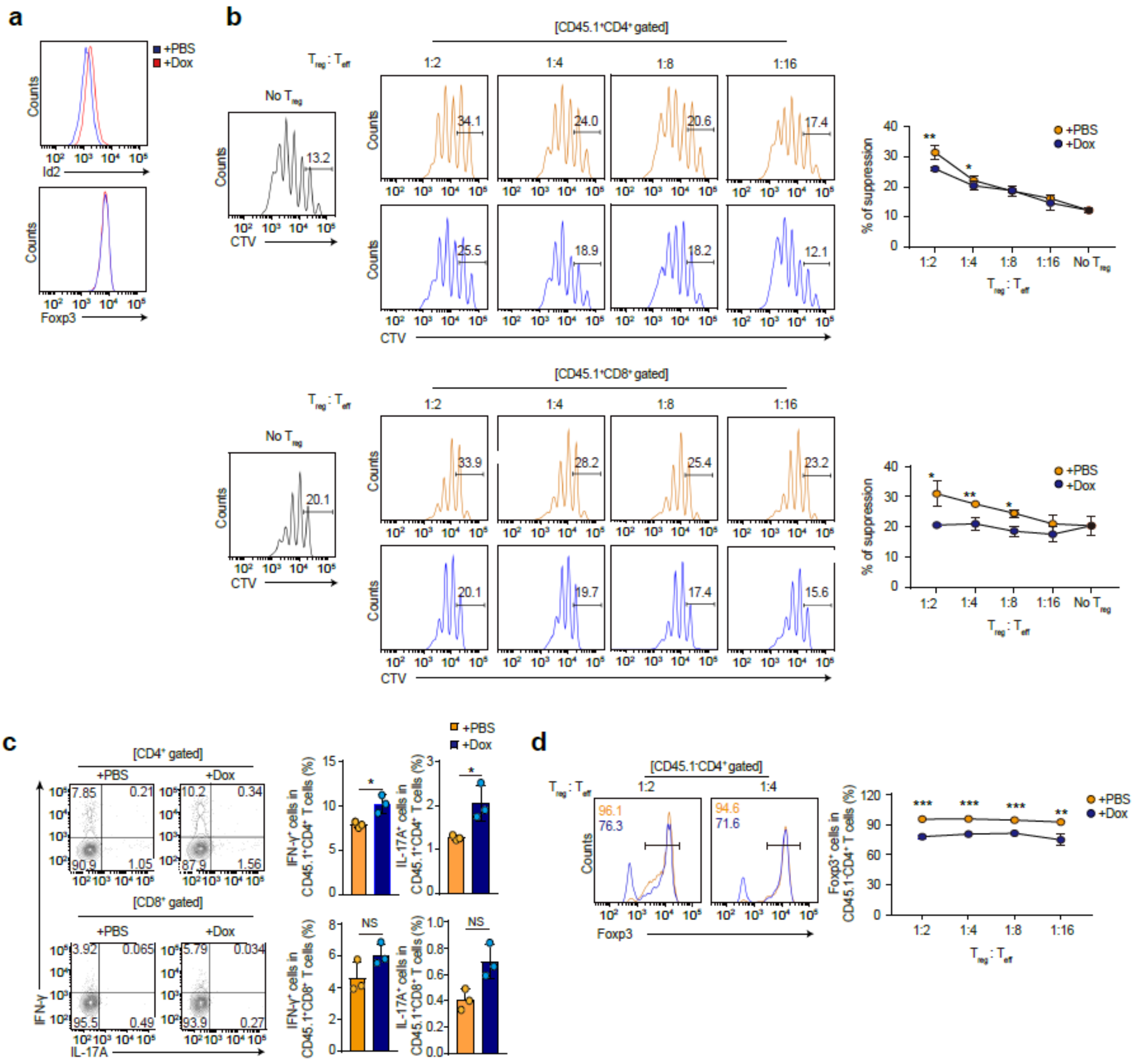

Supplementary Figure 10

Dox-treated Id2 induced T_{reg} cells display reduced suppressive capacity *in vitro.*

a Flow cytometry analysis of Id2 and Foxp3 in $CD4^+Foxp3^+$ T_{reg} cells sorted from PBS- and Dox-treated TetR-*Id2*^{EmGFP}*Foxp3*$^{YFP\text{-}Cre}$ mice. **b** CTV labelled naïve responder $CD45.1^+CD4^+Foxp3^-$ (*upper panel*) and $CD45.1^+CD8^+Foxp3^-$ (*lower panel*) T cells were co-cultured with sorted $CD45.2^+CD4^+Foxp3^+$ T_{reg} cells from PBS- or Dox-treated TetR-*Id2*^{EmGFP}*Foxp3*$^{YFP\text{-}Cre}$ mice, in the presence of APCs purified from T cell depleted splenocytes. Responder T cell proliferation was analyzed by flow cytometry. **c** Intracellular staining of cytokines produced by $CD45.1^+CD4^+Foxp3^-$ (*upper panel*) or $CD45.1^+CD8^+Foxp3^-$ T cells (*lower panel*) that were co-cultured with PBS- or Dox-treated T_{reg} cells at a ratio of 1:4 under conditions similar to (**b**). **d** Flow cytometry analysis of residual Foxp3 expression in T_{reg} cells derived from PBS- or Dox-treated TetR-*Id2*^{EmGFP}*Foxp3*$^{YFP\text{-}Cre}$ mice that were used for suppression assay in (**b**). Data represents three independent experiments. NS=Non-Significant, * $P < 0.05$, ** $P < 0.005$, (Student's *t*-test).

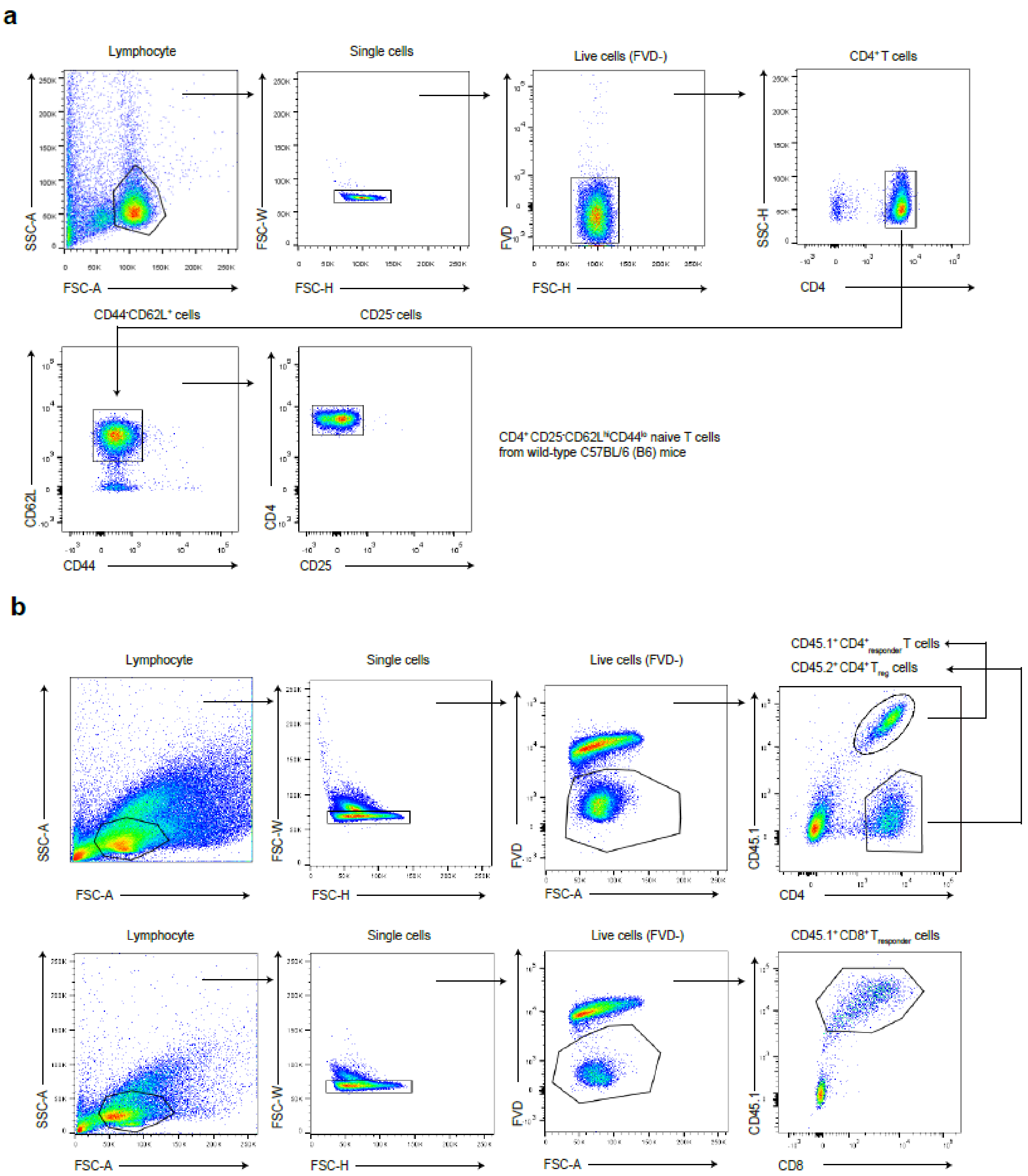

Supplementary Figure 11

Gating strategy for cell sorting

a Gating strategy used in **Figure 1** and **Figure 2** for sorting of CD4$^+$CD25$^-$CD62L^{hi}CD44^{lo} naïve T cells from Wild-type C57BL/6 (B6) mice to generate T$_H$0, T$_H$17, iT$_{reg}$ and ex-Foxp3 T$_H$17 cells *in vitro*. Gating strategies for other experiments were described in each figure. **b** Gating strategy used in **Supplementary Figure 10** for *in vitro* T$_{reg}$ suppression assay.

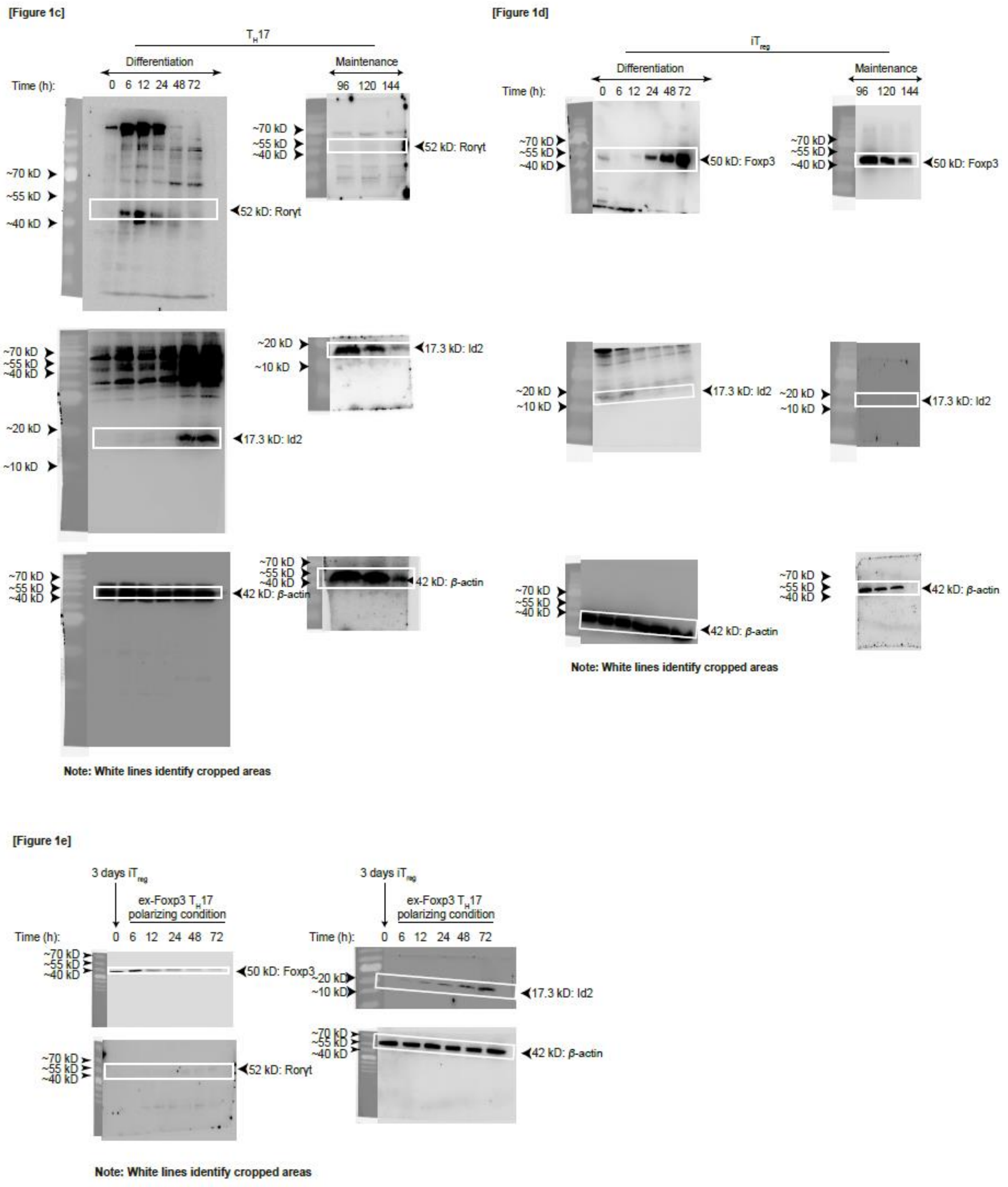

Supplementary Figure 12

Original western blot panels with gel makers for Figures 1c,1d and 1e.

Complete scanned gels for western blots shown in Figure 1c,1d and 1e. Western images are overlaid onto image containing MW markers. White dashed line identifies cropped region shown in respective figure.

Supplementary Table 1: Primer sequences for RT-qPCR

Gene	Primer sequence (5'- 3')	
Hprt	F	TTATGGACAGGACTGAAAGAC
	R	GCTTTAATGTAATCCAGCAGGT
T-bet (TBX21)	F	AGCAAGGACGGCGAATGTT
	R	GGGTGGACATATAAGCGGTTC
Gata3	F	CTCGGCCATTCGTACATGGAA
	R	GGATACCTCTGCACCGTAGC
Rorγt (Rorc)	F	TTTGGAACTGGCTTTCCATC
	R	AAGATCTGCAGCTTTTCCACA
Foxp3	F	TTCCTTCCCAGAGTTCTTCC
	R	CTCAAATTCATCTACGGTCCA
Id2	F	TCATCGATTACATCTTGGACCT
	R	AATTCAGATGCCTGCAAGGA
E2A	F	CATGCCCCCGCCCAG
	R	CAGTTCCTTGTCAGAGCCCA
Ifnγ	F	GAGCCAGATTATCTCTTTCTACC
	R	GTTGTTGACCTCAAACTTGG
Il4	F	CAACGAAGAACACCACAGAG
	R	GGACTTGGACTCATTCATGG
Il5	F	AGCCACTGGATTAGCTCAAGT
	R	GGGGGTCACACATTTGCTTT
Il13	F	GCAACATCACACAGGACCAGA
	R	GTCAGGGAATCCAGGGCTAC
Il17a	F	TTCATCTGTGTCTCTGATGCT
	R	TTCATCTGTGTCTCTGATGCT
Il17f	F	CCCATGGGATTACAACATCACTC
	R	CACTGGGCCTCAGCGATC
Il22	F	GTTGACACTTGTGCGATCTC
	R	CTGATCCTTAGCACTGACTCC
Il10	F	ATAACTGCACCCACTTCCCA
	R	TCATTTCCGATAAGGCTTGG
Tgf-β	F	CTCCCGTGGCTTCTAGTGC
	R	GCCTTAGTTTGGACAGGATCTG
Tnf	F	CCCTCACACTCAGATCATCTTCT
	R	GCTACGACGTGGGCTACAG
