# Supplementary material for: Inflammation-induced Id2 promotes plasticity in regulatory T cells
Source: Nat Commun. 2018 Nov 9;9:4736. doi: 10.1038/s41467-018-07254-2 (PMC6226514; doi:10.1038/s41467-018-07254-2)
Supplement: Supplementary file 3 — Reporting Summary [file 41467_2018_7254_MOESM3_ESM.pdf]

nature research

Corresponding author(s): Dipayan Rudra
Sin-Hyeog Im

Reporting Summary

Nature Research wishes to improve the reproducibility of the work that we publish. This form provides structure for consistency and transparency in reporting. For further information on Nature Research policies, see Authors & Referees and the Editorial Policy Checklist.

Statistical parameters

When statistical analyses are reported, confirm that the following items are present in the relevant location (e.g. figure legend, table legend, main text, or Methods section).

n/a	Confirmed	
☐	☒	The exact sample size (*n*) for each experimental group/condition, given as a discrete number and unit of measurement
☐	☒	An indication of whether measurements were taken from distinct samples or whether the same sample was measured repeatedly
☐	☒	The statistical test(s) used AND whether they are one- or two-sided<br>*Only common tests should be described solely by name; describe more complex techniques in the Methods section.*
☐	☒	A description of all covariates tested
☐	☒	A description of any assumptions or corrections, such as tests of normality and adjustment for multiple comparisons
☐	☒	A full description of the statistics including central tendency (e.g. means) or other basic estimates (e.g. regression coefficient) AND variation (e.g. standard deviation) or associated estimates of uncertainty (e.g. confidence intervals)
☐	☒	For null hypothesis testing, the test statistic (e.g. *F*, *t*, *r*) with confidence intervals, effect sizes, degrees of freedom and *P* value noted<br>*Give P values as exact values whenever suitable.*
☒	☐	For Bayesian analysis, information on the choice of priors and Markov chain Monte Carlo settings
☒	☐	For hierarchical and complex designs, identification of the appropriate level for tests and full reporting of outcomes
☒	☐	Estimates of effect sizes (e.g. Cohen's *d*, Pearson's *r*), indicating how they were calculated
☐	☒	Clearly defined error bars<br>*State explicitly what error bars represent (e.g. SD, SE, CI)*

Our web collection on statistics for biologists may be useful.

Software and code

Policy information about availability of computer code

Data collection	N/A
Data analysis	All statistical analyses were done using GraphPad Prism 7. All FACS data analyses were done using FlowJo (Treestar).

For manuscripts utilizing custom algorithms or software that are central to the research but not yet described in published literature, software must be made available to editors/reviewers upon request. We strongly encourage code deposition in a community repository (e.g. GitHub). See the Nature Research guidelines for submitting code & software for further information.

Data

Policy information about availability of data

All manuscripts must include a data availability statement. This statement should provide the following information, where applicable:

- Accession codes, unique identifiers, or web links for publicly available datasets
- A list of figures that have associated raw data
- A description of any restrictions on data availability

All data that support the findings of this study are available from the corresponding author upon request. The Bisulfite sequencing data was deposited in Gene Expression Omnibus (NCBI) data repository under accession number GEO: GSE118595 [https://www.ncbi.nlm.nih.gov/geo/query/acc.cgi?acc=gse118595].

Field-specific reporting

Please select the best fit for your research. If you are not sure, read the appropriate sections before making your selection.

☒ Life sciences ☐ Behavioural & social sciences ☐ Ecological, evolutionary & environmental sciences

For a reference copy of the document with all sections, see nature.com/authors/policies/ReportingSummary-flat.pdf

Life sciences study design

All studies must disclose on these points even when the disclosure is negative.

Sample size	Sample size is provided in every figure legend.
Data exclusions	No exclusion criteria was used.
Replication	All experiments could successfully be replicated.
Randomization	No randomization method was used.
Blinding	Blinded assessment was performed during histology scoring and weight measurement of animals in colitis experiments.

Reporting for specific materials, systems and methods

Materials & experimental systems

n/a	Involved in the study
☐	☒ Unique biological materials
☐	☒ Antibodies
☐	☒ Eukaryotic cell lines
☒	☐ Palaeontology
☐	☒ Animals and other organisms
☒	☐ Human research participants

Methods

n/a	Involved in the study
☒	☐ ChIP-seq
☐	☒ Flow cytometry
☒	☐ MRI-based neuroimaging

Unique biological materials

Policy information about availability of materials

Obtaining unique materials	All the materials used are available without restrictions.

Antibodies

Antibodies used	The information is provided in Methods section.
Validation	All the antibodies used have validation statement provided by their corresponding manufacturers that are available online.

Eukaryotic cell lines

Policy information about cell lines

Cell line source(s)	ATCC
Authentication	Yes, we check phenotypic markers by flow cytometry and functional studies.
Mycoplasma contamination	Mycoplasma were not detected.
Commonly misidentified lines (See ICLAC register)	N/A

Animals and other organisms

Policy information about studies involving animals; ARRIVE guidelines recommended for reporting animal research

Laboratory animals	Added in Methods section and corresponding figure legends.
Wild animals	This study does not involve any wild animal.
Field-collected samples	This study does not involve any sample collected from field.

Flow Cytometry

Plots

Confirm that:

- [x] The axis labels state the marker and fluorochrome used (e.g. CD4-FITC).
- [x] The axis scales are clearly visible. Include numbers along axes only for bottom left plot of group (a 'group' is an analysis of identical markers).
- [x] All plots are contour plots with outliers or pseudocolor plots.
- [x] A numerical value for number of cells or percentage (with statistics) is provided.

Methodology

Sample preparation	Provided in Methods section.
Instrument	Provided in Methods section.
Software	Provided in Methods section.
Cell population abundance	Provided in Methods section.
Gating strategy	Gating strategies are provided in Supplementary Fig. 12 and 13.

- [x] Tick this box to confirm that a figure exemplifying the gating strategy is provided in the Supplementary Information.
